# 

**Published:** 1856-11

**Authors:** 


### CONTENTS.

### *ORIGINAL COMMUNICATIONS.*

PAGE

ARTICLE I.—A Thesis on Acute Inflammation, submitted to the Faculty of the Atlanta Medical College, Session 1856. By C. C. LLOYD, of Wetumpka, Alabama, ........ 129

ARTICLE II.—What is Hereditary Transmission? By J. J. M. GOSS, M. D., Jackson County, Georgia., ........ 136

ARTICLE III.—Causes of Diseases. By Dr. W. T. GRANT, Wrightsboro', Columbia County, Georgia, ........ 141

ARTICLE IV.—Abuse of Remedial Agents. By J. G. WESTMORELAND, M. D., of Atlanta, Georgia, ........ 145

ARTICLE V.—Puerperal Fever—Its Pathology. By J. BORING, M. D., Professor of Obstetrics and Diseases of Women and Children in the Atlanta Medical College, ........ 148

### *SELECTIONS.*

The Abnormal Conditions of the Kidneys in Bright's Disease........ 158

Remarks on Vesico-Vaginal Fistule,........ 172

On the Constitutional Effects of Anæsthetic Agents,........ 174

Female Medical Education,........ 179

### *EDITORIAL AND MISCELLANEOUS.*

To Subscribers,........ 183

Death of Dr. G. M. Scarlett,........ 184

Menstruation in Old Age,........ 184

Bibliographical,........ 185

Flint on "Abnormal Condition of the Kidneys in Bright's Disease,........ 188

A Chapter Omitted in most Treatises on General Pathology,........ 189

Editorial Correspondence,........ 192

